# Supplementary material for: Scalable telomere-to-telomere assembly for diploid and polyploid genomes with double graph
Source: ArXiv. 2023 Jun 6:arXiv:2306.03399v1. Preprint. [Version 1] (PMC10274930)
Supplement: Supplement 1 [file NIHPP2306.03399v1-supplement-1.pdf]

arXiv:2306.03399v1 [q-bio.GN] 6 Jun 2023

Supplementary Information for

"Scalable telomere-to-telomere assembly for diploid and polyploid genomes with double graph"

1 Software commands

1.1 Filtering ultra-long reads

We discarded short ultra-long reads to avoid assembly errors and reduce the running time. For HPRC Year 2 samples, ultra-long reads with a length less than 100kb were filtered out using seqkit (version 2.3.0):

```
seqkit seq -m1000000 <ultra-long-reads.fasta>
```

In the case of HPRC Year 1 and plant samples, ultra-long reads shorter than 50kb were removed.

```
seqkit seq -m500000 <ultra-long-reads.fasta>
```

1.2 Hifiasm

To produce Hi-C phased assemblies with HiFi, Hi-C and ultra-long reads, hifiasm (version 0.19.4-r587) was run with the following command:

```
hifiasm -o <outputPrefix> -t <nThreads> --h1 <HiC-reads-R1.fasta> --h2 <HiC-reads-R2.fasta> \
--hom-cov <homozygous_coverage> --ul <ultra-long-reads.fasta> <HiFi-reads.fasta>
```

For the trio-binning assembly, we first built the paternal trio index and the maternal trio index by yak (version 0.1-r62-dirty) with the following commands:

```
yak count -b37 -t <nThreads> -o <pat.yak> <paternal-short-reads.fastq>
yak count -b37 -t <nThreads> -o <mat.yak> <maternal-short-reads.fastq>
```

and then we produced the paternal assembly and the maternal assembly with the following command:

```
hifiasm -o <outputPrefix> -t <nThreads> -1 <pat.yak> -2 <mat.yak> --ul <ultra-long-reads.fasta> \
--hom-cov <homozygous_coverage> <HiFi-reads.fasta>
```

To assemble the haploid Arabidopsis genome with HiFi and ultra-long reads, hifiasm was run with:

```
hifiasm -o <outputPrefix> -t <nThreads> -l0 --ul <ultra-long-reads.fasta> <HiFi-reads.fasta>
```

We also produced the HiFi-only assembly of Arabidopsis using:

```
hifiasm -o <outputPrefix> -t <nThreads> -l0 <HiFi-reads.fasta>
```

For the autotetraploid potato genome, hifiasm was run with HiFi and ultra-long reads along with the genetic map using the command lines as follows:

```
hifiasm -o <outputPrefix> --hom-cov 116 -D10 -t <nThreads> -5 <genetic-map> \
--ul <ultra-long-reads.fasta> <HiFi-reads.fasta>
```

Without ultra-long reads, the command lines are:

```
hifiasm -o <outputPrefix> --hom-cov 116 -D10 -t <nThreads> -5 <genetic-map> <HiFi-reads.fasta>
```

1.3 Verkko

For the haploid genome assembly of Arabidopsis, Verkko (version 1.3.1) was run with the following command line:

```
verkko -d <outDir> --hifi <HiFi-reads.fasta> --nano <ultra-long-reads.fasta>
```

For the trio-binning assembly, Verkko was run with:

```
verkko -d <outDir> --hifi <HiFi-reads.fasta> --nano <ultra-long-reads.fasta> \
   --hap-kmers <mat_hapmer_db> <pat_hapmer_db> trio
```

We also ran Verkko with gfase to assemble human genomes with Hi-C reads. Its WDL workflow is started by creating an unphased assembly graph with Verkko: https://dockstore.org/workflows/github.com/human-pangenomics/hpp_production_workflows/VerkkoCreateUnphasedGFA:master?tab=info, and then phases with the HiC reads on top of the assembly graph using gfase: https://dockstore.org/workflows/github.com/meredith705/gfase_wdl/gfaseWorkflow:main?tab=info. The final step is to rerun Verkko with the phasing results produced by gfase: https://github.com/human-pangenomics/hpp_production_workflows/blob/master/assembly/wdl/tasks/verkko_consensus_from_gfase.wdl.

1.4 Running with Terra using cloud computing

We performed the assemblies of human genomes using preemptible instances provided by the Google Cloud Platform. Hifiasm (UL) was divided into three steps to make full use of the preemptible instances. Step 1 of hifiasm (UL) with trio-binning is:

```
hifiasm -o <outputPrefix> -t <nThreads> --bin-only -1 <pat.yak> -2 <mat.yak> \
--hom-cov <homozygous_coverage> <HiFi-reads.fasta>
```

Step 2 of hifiasm (UL) with trio-binning is:

```
hifiasm -o <outputPrefix> -t <nThreads> --bin-only -1 <pat.yak> -2 <mat.yak> \
--hom-cov <homozygous_coverage> --ul <ultra-long-reads.fasta> <any_temporary_file>
```

Step 3 of hifiasm (UL) with trio-binning is:

```
hifiasm -o <outputPrefix> -t <nThreads> -1 <pat.yak> -2 <mat.yak> \
--hom-cov <homozygous_coverage> --ul <ultra-long-reads.fasta> <any_temporary_file>
```

For the single-sample phased assembly with Hi-C reads, the first step of hifiasm (UL) is:

```
hifiasm -o <outputPrefix> -t <nThreads> --bin-only --hom-cov <homozygous_coverage> <HiFi-reads.fasta>
```

The second step of hifiasm (UL) with Hi-C reads is:

```
hifiasm -o <outputPrefix> -t <nThreads> --bin-only --hom-cov <homozygous_coverage> \
--ul <ultra-long-reads.fasta> <any_temporary_file>
```

The final step of hifiasm (UL) with Hi-C reads is:

```
hifiasm -o <outputPrefix> -t <nThreads> --hom-cov <homozygous_coverage> \
--h1 <HiC-reads-R1.fasta> --h2 <HiC-reads-R2.fasta> --ul <ultra-long-reads.fasta> <any_temporary_file>
```

For the trio-binning assembly of Verkko, the first step is producing an unphased assembly graph with the following WDL script:

```
https://dockstore.org/workflows/github.com/human-pangenomics/hpp_production_workflows/VerkkoAssemb
lyScatter:master?tab=info
```

And then the trio phasing was added by calling Verkko again with Meryl hapmer DBs:

```
https://dockstore.org/workflows/github.com/human-pangenomics/hpp_production_workflows/TrioVerkkoAs
semblyScatter:master?tab=info
```

where hapmer DBs were created from parental Illumina data with:

```
https://dockstore.org/workflows/github.com/human-pangenomics/hpp_production_workflows/Meryl:
master?tab=info
```

Similarly, the Hi-C phased assembly of Verkko was started by creating an unphased assembly graph:

```
https://dockstore.org/workflows/github.com/human-pangenomics/hpp_production_workflows/VerkkoCreate
UnphasedGFA:master?tab=info
```

Then we ran gfase with the Hi-C reads to produce Hi-C phasing information:

```
https://dockstore.org/workflows/github.com/meredith705/gfase_wdl/gfaseWorkflow:main?tab=info
```

The phasing results from gfase were finally integrated into Verkko in a WDL:

```
https://github.com/human-pangenomics/hpp_production_workflows/blob/master/assembly/wdl/tasks/verkk
o_consensus_from_gfase.wdl
```

1.5 Running asmgene

For human genome assemblies, we aligned the cDNAs to the CHM13v2 reference genome and assembled contigs by minimap2 (version 2.24-r1122), and evaluated the gene completeness with paftools.js from the minimap2 package:

```
minimap2 -cxsplice:hq -t <nThreads> <ref.fa> <cDNAs.fa> > <ref.paf>
minimap2 -cxsplice:hq -t <nThreads> <asm_contig.fa> <cDNAs.fa> > <asm.paf>
paftools.js asmgene -a -i.97 <ref.paf> <asm.paf>
```

1.6 BUSCO

For non-human genome assemblies, BUSCO (version 5.4.4) was used with the following command:

```
busco -i <asm.fa> -m genome -o <outDir> -c <nThreads> -l <lineage_dataset>
```

where 'lineage_dataset' was set to *brassicales_odb10* and *solanales_odb10* for Arabidopsis and potato genome assemblies, respectively.

1.7 Phasing accuracy evaluation

For human genome assemblies, we used yak (version 0.1-r62-dirty) to measure the hamming error rate and the switch error rate:

```
yak trioeval -t <nThreads> <paternal.yak> <maternal.yak> <asm_contig.fa>
```

For the potato genome assembly, we employed haplotype-specific HiFi reads as markers to assess the phasing errors.

1.8 Counting Telomere-to-Telomere (T2T) contigs

The HPRC workflow (https://github.com/biomonika/HPP/blob/main/assembly/wdl/workflows/assessAsemblyCompletness.wdl) was utilized to detect the T2T contigs. The CHM13v2 reference was set as the reference genome when running the HPRC workflow with human genome assemblies. For non-human Arabidopsis and potato genomes, all assemblies were aligned to the published genomes generated from the same datasets.

Supplementary Table 1: Phasing errors of HPRC Year 2 and potato assemblies

Dataset	Assembler	Phasing error (switch/hamming)				
		Hap1 (%)	Hap2 (%)	Hap3 (%)	Hap4 (%)	Unassigned (%)
HG002	hifiasm(UL)_trio	0.20/0.16	0.29/0.27	/	/	/
	verkko_trio	0.17/0.13	0.23/0.22	/	/	/
	hifiasm(UL)_hic	0.19/0.23	0.28/0.24	/	/	/
	verkko_gfase	0.26/0.32	0.19/0.19	/	/	0.02/0.02
HG01099	hifiasm(UL)_trio	0.46/0.47	0.62/0.70	/	/	/
	verkko_trio	0.46/0.49	0.60/0.46	/	/	/
	hifiasm(UL)_hic	0.43/0.38	0.67/0.76	/	/	/
	verkko_gfase	0.74/0.69	0.74/0.70	/	/	0.04/0.03
HG02004	hifiasm(UL)_trio	0.94/1.05	0.69/0.52	/	/	/
	verkko_trio	0.87/0.95	0.63/0.46	/	/	/
	hifiasm(UL)_hic	0.87/0.96	0.74/0.83	/	/	/
	verkko_gfase	0.70/0.71	0.80/0.89	/	/	0.89/0.90
HG02071	hifiasm(UL)_trio	0.37/0.30	0.76/0.65	/	/	/
	verkko_trio	0.34/0.28	0.68/0.64	/	/	/
	hifiasm(UL)_hic	0.40/0.44	0.64/1.43	/	/	/
	verkko_gfase	0.63/0.98	0.60/0.54	/	/	0.05/0.05
HG02293	hifiasm(UL)_trio	0.73/0.59	1.14/1.31	/	/	/
	verkko_trio	1.08/1.04	0.67/0.52	/	/	/
	hifiasm(UL)_hic	0.82/1.44	1.00/1.24	/	/	/
	verkko_gfase	0.84/1.52	0.90/0.87	/	/	0.59/0.47
HG02300	hifiasm(UL)_trio	0.92/0.81	0.77/0.67	/	/	/
	verkko_trio	0.84/0.72	0.70/0.59	/	/	/
	hifiasm(UL)_hic	0.88/0.84	0.77/0.70	/	/	/
	verkko_gfase	0.87/1.30	0.69/0.65	/	/	0.35/0.43
HG02647	hifiasm(UL)_trio	0.37/0.31	0.53/0.43	/	/	/
	verkko_trio	0.35/0.64	0.50/0.87	/	/	/
	hifiasm(UL)_hic	0.38/0.50	0.49/1.29	/	/	/
	verkko_gfase	0.54/1.51	0.53/1.61	/	/	0.05/0.04
HG02809	hifiasm(UL)_trio	0.49/0.58	0.55/0.41	/	/	/
	verkko_trio	0.49/0.61	0.57/0.53	/	/	/
	hifiasm(UL)_hic	0.50/0.53	0.55/0.77	/	/	/
	verkko_gfase	0.49/0.64	0.57/0.72	/	/	1.09/1.18
HG03710	hifiasm(UL)_trio	0.27/0.26	0.75/0.73	/	/	/
	verkko_trio	0.23/0.23	0.69/0.68	/	/	/
	hifiasm(UL)_hic	0.40/0.36	0.54/0.53	/	/	/
	verkko_gfase	0.51/0.54	0.53/0.55	/	/	0.06/0.19
HG03927	hifiasm(UL)_trio	1.02/1.01	0.51/0.44	/	/	/
	verkko_trio	0.91/0.91	0.40/0.37	/	/	/
	hifiasm(UL)_hic	0.87/1.27	0.64/0.80	/	/	/
	verkko_gfase	0.83/1.41	0.51/1.00	/	/	0.25/0.19
HG04228	hifiasm(UL)_trio	0.78/0.67	0.69/0.63	/	/	/
	verkko_trio	0.73/0.63	0.62/0.49	/	/	/
	hifiasm(UL)_hic	0.83/0.77	0.67/0.64	/	/	/
	verkko_gfase	0.94/0.78	0.77/0.63	/	/	0.09/0.11
Potato ($\geq$500kb)	hifiasm(UL)	0.08/0.40	0.14/0.92	0.12/0.96	0.20/2.04	/
	hifiasm(HiFi)	0.04/0.26	0.08/0.59	0.07/0.59	0.12/1.27	/

The phasing switch error rate refers to the proportion of adjacent haplotype-specific marker pairs originating from different haplotypes, while the phasing hamming error rate represents the percentage of haplotype-specific markers that are incorrectly phased. For human genome assemblies, phasing errors were calculated using haplotype-specific 31-mers obtained from parental short reads with yak. For the potato genome assembly, we employed haplotype-specific HiFi reads as markers to assess the phasing errors.

Supplementary Table 2: Phasing errors of HPRC Year 1 assemblies

Dataset	Assembler	Phasing error (switch/hamming)				
		Hap1 (%)	Hap2 (%)	Hap3 (%)	Hap4 (%)	Unassigned (%)
HG00438	hifiasm(UL)_trio	1.07/0.89	1.10/1.02	/	/	/
	verkko_trio	1.00/1.09	1.00/1.03	/	/	/
HG00741	hifiasm(UL)_trio	1.11/1.27	0.59/0.49	/	/	/
	verkko_trio	0.54/0.37	1.03/1.26	/	/	/
HG01175	hifiasm(UL)_trio	1.03/0.83	0.87/0.71	/	/	/
	verkko_trio	0.78/0.73	0.93/0.87	/	/	/
HG03516	hifiasm(UL)_trio	0.64/0.44	0.83/0.79	/	/	/
	verkko_trio	0.62/0.49	0.79/0.86	/	/	/
HG01071	hifiasm(UL)_trio	0.39/0.36	0.92/1.06	/	/	/
	verkko_trio	0.87/0.98	0.33/0.42	/	/	/
HG01978	hifiasm(UL)_trio	0.92/0.75	0.89/0.92	/	/	/
	verkko_trio	0.81/0.95	0.86/0.99	/	/	/
HG00733	hifiasm(UL)_trio	1.00/0.83	1.02/1.07	/	/	/
	verkko_trio	0.92/1.03	0.91/0.75	/	/	/
HG00621	hifiasm(UL)_trio	0.45/0.55	0.73/0.58	/	/	/
	verkko_trio	0.61/0.45	0.41/0.53	/	/	/
HG01891	hifiasm(UL)_trio	0.70/0.70	0.59/0.42	/	/	/
	verkko_trio	0.67/0.70	0.57/0.65	/	/	/
HG01106	hifiasm(UL)_trio	0.34/0.30	0.45/0.37	/	/	/
	verkko_trio	0.38/0.46	0.31/0.34	/	/	/
HG02886	hifiasm(UL)_trio	0.85/0.86	0.27/0.27	/	/	/
	verkko_trio	0.26/0.49	0.83/1.30	/	/	/

The phasing switch error rate refers to the proportion of adjacent haplotype-specific marker pairs originating from different haplotypes, while the phasing hamming error rate represents the percentage of haplotype-specific markers that are incorrectly phased. Phasing errors were calculated using haplotype-specific 31-mers obtained from parental short reads with yak.

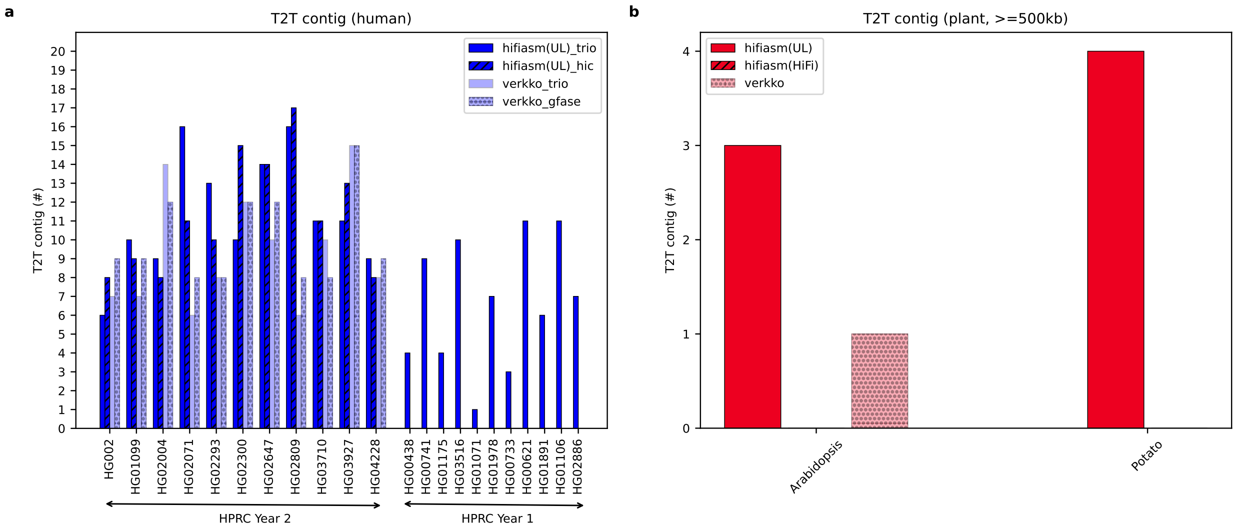

Supplementary Fig. 1: Numbers of Telomere-to-Telomere (T2T) contigs for different assemblies. A contig is considered as a T2T contig only if it can be aligned to an entire chromosome of the reference genome with telomeres on both ends. We utilized the Human Pangenome Reference Consortium (HPRC) workflow to detect the T2T contigs. **(a)** Numbers of telomere-to-telomere contigs for human genomes. There is no T2T contig for HPRC Year 1 assemblies of Verkko. All assemblies were aligned to the CHM13v2 reference genome to detect T2T contigs. **(b)** Numbers of telomere-to-telomere contigs for plant genomes. To detect T2T contigs, all assemblies were aligned to the published genomes generated from the same datasets.

Supplementary Table 3: Statistics of human datasets

Group	Sample	Sex	HiFi read	Ultra-long read	
			coverage	coverage (≥50kb)	coverage (≥100kb)
HPRC Year 1	HG00438	Female	30X	29X	16X
	HG00741	Female	38X	33X	19X
	HG01175	Female	36X	32X	18X
	HG03516	Female	35X	30X	17X
	HG01071	Female	35X	29X	16X
	HG01978	Female	37X	28X	16X
	HG00733	Female	33X	25X	5X
	HG00621	Male	40X	25X	14X
	HG01891	Female	37X	26X	16X
	HG01106	Male	48X	29X	15X
	HG02886	Female	43X	24X	14X
HPRC Year 2	HG002	Male	43X		31X
	HG01099	Male	39X		31X
	HG02004	Female	43X		45X
	HG02071	Male	36X		24X
	HG02293	Female	38X		40X
	HG02300	Female	35X		36X
	HG02647	Male	46X		21X
	HG02809	Female	43X		34X
	HG03710	Male	36X		26X
	HG03927	Female	45X		29X
	HG04228	Male	49X		30X

All HPRC Year 2 assemblies were produced using ultra-long reads with a minimum length of 100 kb. As there are not enough ≥100 kb ultra-long reads, HPRC Year 1 assemblies were generated with ultra-long reads with a minimum length of 50 kb.

Supplementary Table 4: Numbers of Telomere-to-Telomere (T2T) contigs and scaffolds for human genome assemblies of Verkko

Group	Sample	verkko_trio		verkko_gfase	
		T2T Contig	T2T Scaffold	T2T Contig	T2T Scaffold
HPRC Year 1	HG00438	0	1		
	HG00741	0	5		
	HG01175	0	5		
	HG03516	0	2		
	HG01071	0	4		
	HG01978	0	10		
	HG00733	0	2		
	HG00621	0	12		
	HG01891	0	10		
	HG01106	0	15		
	HG02886	0	6		
HPRC Year 2	HG002	7	8	9	6
	HG01099	7	10	9	9
	HG02004	14	11	12	12
	HG02071	6	21	8	17
	HG02293	8	13	8	14
	HG02300	12	11	12	8
	HG02647	10	15	12	13
	HG02809	6	15	8	15
	HG03710	10	8	8	10
	HG03927	15	9	15	9
	HG04228	8	15	9	11
